# Management of Optic Pathway Glioma: A Systematic Review and Meta-Analysis

**DOI:** 10.3390/cancers14194781

**Published:** 2022-09-30

**Authors:** Omid Yousefi, Pouria Azami, Mohammadmahdi Sabahi, Rocco Dabecco, Badih Adada, Hamid Borghei-Razavi

**Affiliations:** 1 Trauma Research Center, Shiraz University of Medical Sciences, Shiraz 71348, Iran; 2 Neurosurgery Research Group (NRG), Student Research Committee, Hamadan University of Medical Sciences, Hamadan 65141, Iran; 3 Department of Neurological Surgery, Pauline Braathen Neurological Center, Cleveland Clinic Florida, Weston, FL 33331, USA

**Keywords:** optic pathway glioma, surgery, radiotherapy, chemotherapy, outcome, systematic review, meta-analysis

## Abstract

**Simple Summary:**

In the current study, we systematically reviewed the literature regarding the management of optic pathway glioma (OPG). This review will analyze demographics, information regarding the type of treatment, radiological and clinical (visual) outcomes, and also complications. Of the 105 eligible studies, a total of 4177 patients underwent different therapeutic modalities, including surgery, radiotherapy, and chemotherapy. The mean age was 8.17 years, and the gender ratio was 97 males per 100 females. Based on the available data, the mean follow-up was 98.6 months, and 48.7% of the patients were known cases of neurofibromatosis type1. According to the Dodge classification, 1104, 941, and 1353 tumors were D_1_, D_2_, and D_3_, respectively. The toxicity induced by either chemo- or radiotherapy was the most common complication, followed by ophthalmologic complications rather than visual acuity exacerbation. Small sample sizes in studies that compare different therapeutic modalities for OPG treatment, heterogeneity in multiple parameters including tumor locations, patients’ age distribution who received treatment, patients lost to follow-up in many studies, lack of high-power studies, and insufficient power to adequately prove the efficacy or safety profile of different therapeutic modalities are all possible limitations of this systematic review. Moreover, in order to reduce the versatility between studies, we have considered all different types of surgical procedures, distinct chemotherapy (CT) regimens, and separate ways to deliver irradiation in OPG patients as surgery, CT, and radiotherapy (RT), respectively. This assumption was made since available data were neither representative of actual therapeutic modalities nor provided an opportunity to conduct sub-group analysis. Additionally, since children’s visual assessments are notoriously recalcitrant, it might be obscure the results of visual acuity in pediatrics.

**Abstract:**

Background: OPG accounts for 3–5% of childhood central nervous system (CNS) tumors and about 2% of pediatric glial lesions. Methods: Article selection was performed by searching PubMed, Web of Science, and Cochrane databases. Results: The pooled mortality rate was 0.12 (95%CI 0.09–0.14). Due to the unrepresentative data, improved and not changed outcomes were classified as favorable outcomes and worsened as unfavorable. Meta-analyses were performed to determine the rate of clinical and radiological favorable outcomes. In terms of visual assessment, the pooled rate of a favorable outcome in chemotherapy, radiotherapy, and surgery was 0.74, 0.81, and 0.65, respectively, and the overall pooled rate of the favorable outcome was 0.75 (95%CI 0.70–0.80). In terms of radiological assessment, the rate of a favorable outcome following chemotherapy, radiotherapy, and surgery was 0.71, 0.74, and 0.67, respectively, and the overall pooled rate of the favorable outcome is 0.71 (95%CI 0.65–0.77). The subgroup analysis revealed no significant difference in the rate of clinical and radiological favorable outcomes between the different treatment modalities (*p* > 0.05). Conclusion: Our analyses showed that each therapeutic modality represents viable treatment options to achieve remission for these patients.

## 1. Introduction

Optic pathway glioma (OPG) accounts for 3–5% of childhood central nervous system (CNS) tumors and about 2% of pediatric glial lesions [1,2]. Compared to adults, children under the age of 10 have a higher prevalence of OPG [3]. Any location along the optic tract or hypothalamus can be affected and as expected, the majority of patients present with visual and ophthalmological disorders. However, endocrinopathy and hydrocephalus-related symptoms, due to the mass effect of the lesion, can be anticipated [4]. OPG is believed to be the most prevalent intracranial tumor in patients with neurofibromatosis type 1 (NF-1) and can occur in 15–20% of NF-1 cases [5,6]. Some chromosomal abnormalities, notably deletion of chromosome 17q and neurofibromin (in NF-1 patients), have been regarded as the underlying etiology of this tumor [7].

Due to the aggressive behavior of the OPG, treatment should be taken into consideration in most cases. However, in asymptomatic patients, observation and serial imaging is considered the standard of care, as there have been reports in the literature of spontaneous regression of the mass [8]. In symptomatic patients, surgical resection or debulking of the mass, chemotherapy (CT), and radiotherapy (RT) are among the interventions used for the treatment of OPG [7]. Much is not known precisely about the distribution of OPGs pathology, location and outcome, and complications of the treatments. In this systematic review and meta-analysis, we conducted a comprehensive search of articles reflecting outcomes of the OPG patients after mentioned treatment modalities to assess the characteristics of the patients and tumor and the following outcomes.

## 2. Materials and Methods

### 2.1. Search Strategy

A comprehensive search, using the Preferred Reporting Items for Systematic Reviews and Meta-Analyses (PRISMA) guidelines [9], was conducted to include the studies, reflecting the outcomes and complications of the OPG treatment. The systematic literature search was performed by using PubMed, Web of Science, and Cochrane databases to include the studies published prior to April 2022. “Optic pathway glioma”, “Radiotherapy”, “Chemotherapy”, and “Surgical resection” and their synonym terms, were the keywords used for the search.

### 2.2. Eligibility Criteria

All of the studies reflecting the outcomes associated with the treatment of the OPG were included in the current review, except those that (1) were not written in English (2) were published prior to 1980 (3) were in the forms of editorial, case report, commentary, and letter to the editors; (4) reflected any type of treatment, rather than CT, RT, and surgical intervention; (5) included less than 3 patients.

The obtained studies were imported into an EndNote library (EndNote X9.1.1, Thomson Reuters) and after the removal of duplicates, the initial screening was carried out by evaluation of the title and the abstracts by two authors (O.Y and P.A). All the remaining relevant articles were reviewed in the full text by two authors (M.S and P.A). Disagreements were settled by discussion, and if necessary, the senior author’s (H.B) viewpoint was sought.

**Figure 1:**
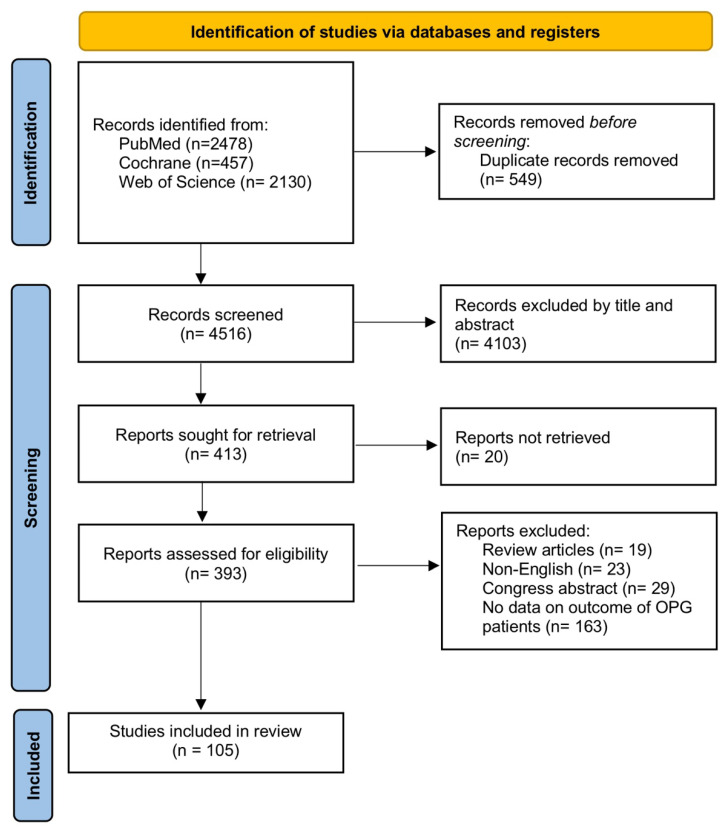
PRISMA flowchart illustrating the search and selection process of included articles.

This query identified 4516 papers that were assessed for relevance by two independent reviewers (O.Y. and P.A.). Disagreements were resolved by a third author (M.S). The initial search identified 2130 papers in the Web of sciences, 2478 papers in MEDLINE (PubMed), and 457 papers in Cochrane. After removing 549 duplicate papers, titles and abstracts of 4516 records were screened, of which 4103 records were irrelevant. Finally, 413 papers were selected and surveyed for eligibility. Out of 413 records, 145 articles were excluded, including 19 review articles, 23 non-English, 29 congress abstracts, 30 commentary, 17 letters to the editor, and 25 articles with unavailable full texts. From the 268 remaining articles, 163 studies were removed as they did not include data on outcomes of different therapeutic modalities in OPG patients. Finally, 105 papers were found eligible for the systematic review (Figure 1).

### 2.3. Data Extraction

The following items were extracted from the eligible studies by two reviewers (M.S and P.A): (1) number of the cases, (2) mean age, (3) male to female ratio, (4) history of NF-1, (5) symptoms, (6) the treatment strategy, (7) previous treatments, (8) further salvage therapies, (9) pathology of the tumor, (10) location of the tumor, (11) number and causes of the mortalities, (12) complications associated with the treatments, (13) visual outcome, and (14) radiological outcome.

### 2.4. Risk of Bias

Assessment of the risk of bias was conducted by using the national institute of health (NIH) tool based on the articles’ type (accessed on 14 December 2021) [10]. Two authors (P.A and M.S) evaluated the bias risk and if any conflict was noted, it was discussed with another author (O.Y).

### 2.5. Statistical Analysis

The descriptive outcomes are presented as the frequency (Percentage) for categorical variables, and as the mean ± standard deviation for continuous variables. In cases where the patients’ age or the follow-up duration were presented as the median and range, it was transformed to the mean and standard deviation, using the method proposed by Hozo et al. [11].

The meta-analysis was performed on the clinical (visual) and radiological outcomes. In order to consider the heterogenicity of the settings of the different studies, the random-effects model was applied. The pooling was conducted using RStudio software version 1.2.5042 (RStudio Team (2020). RStudio: Integrated Development for R. RStudio, Inc., Boston, MA) and Meta package v4.17-0 [12]. The pooled rates are shown in detail in forest plots, as estimated proportion points with a 95% confidence interval. The Heggin’s index (I^2^) was used to evaluate the between study heterogenicity. I^2^ higher than 50% was regarded as high between studies heterogenicity and to resolve this issue, a sensitivity test, by removal of the outlier studies was conducted and analysis was performed without outlier studies.

The probable publication bias was assessed by using Egger’s test and the Funnel plots, and in case of significant bias, the trim and fill method and Rucker limited meta-analysis were applied to compensate for the bias [13].

## 3. Results

Initially, 4516 articles were attained from the systematic search and after the removal of the duplicates, the preliminary screening was conducted based on the title and abstracts of the 3600 manuscripts. Following the full-text review of the 415 articles, 105 articles were found eligible according to our criteria [14,15,16,17,18,19,20,21,22,23,24,25,26,27,28,29,30,31,32,33,34,35,36,37,38,39,40,41,42,43,44,45,46,47,48,49,50,51,52,53,54,55,56,57,58,59,60,61,62,63,64,65,66,67,68,69,70,71,72,73,74,75,76,77,78,79,80,81,82,83,84,85,86,87,88,89,90,91,92,93,94,95,96,97,98,99,100,101,102,103,104,105,106,107,108,109,110,111,112,113,114,115,116,117,118]. The data extraction was carried out based on the available data of the articles and for each variable, only studies that reported that specific variable were considered.

According to the extracted data, the aforementioned characteristics of the 4177 patients were evaluated. The mean age was 8.17 years (range: 0 to 85 years) and among all included articles, 85 articles have detailed distribution of age. Out of these 85 articles, 72, 2, and 11 articles reported data on pediatrics (age < 18 years), adults (age ≥ 18 years), and mixture of pediatric and adults, respectively. Data pooling on studies with focus on pediatrics showed that there were 3000 OPG patients in this group with mean age of 5.20 years (range 0–18 years), while there were 54 adult OPG patients with mean age of 33.56 years (range 18–68 years). In addition, the male: female ratio was 0.97 among the patients. The mean follow-up duration among the 2930 patients with documented reports was also 98.6 months (range: 15 days to 32 years).

### 3.1. Neurofibromatosis Type 1

Based on the available data of the 3221 patients, 48.7% of the patients were known cases of the NF-1.

### 3.2. Tumor Location

The location of the lesions was categorized using the Dodge classification [119]. Additionally, reports that did not use the Dodge classification format were categorized using the Dodge classification. According to the available data of the 3628 tumors, 1104 were D1, 941 were D2, and 1353 were D3. The remaining 230 patients’ records were not described in detail; however, 40 of them had tumors in D1 or D2, while the other 190 had tumors in D2 or D3.

### 3.3. Pathology of the Tumor

The tumor’s pathology was classified using the world health organization (WHO) classification of CNS tumors [120]. Of the 1706 lesions which had a reported pathology, 1141(66%) cases were categorized as G1, 282 (16.5%) cases as G2, 16 (less than 1%) cases as G3, and 11 (less than 1%) cases as G4. The pathology of the 249 (14.5%) patients was described as low-grade glioma (G1 or G2) and 7 (less than 1%) patients as high-grade glioma.

### 3.4. Symptoms

Based on the available data of the 3322 patients, patients with OPG mainly present with visual deterioration. In our survey, the disorders related to the optic disk were categorized in the optic nerve disorders group. Diplopia, photophobia, color vision defect, and eye movement disorders were also categorized in the other ophthalmologic group. The symptoms and their frequencies are illustrated in Table 1.

### 3.5. Complications

About 84 articles published records of the 3405 patients with documented adverse events associated with the treatments. The toxicity induced by chemo- or radiotherapy was the most common, followed by the ophthalmologic complications (eye-related complications rather than visual acuity exacerbation). The details of the complications and their frequencies are shown in Table 2.

### 3.6. Mortality

Mortality events were documented in 44 studies, which included 2611 patients. The pooled mortality rate was 0.12 (95% confidence interval (CI): 0.09; 0.14, I^2^ = 56.5%). The pooled rate of mortality after removing the 4 outlier heterogeneous studies was 0.1153 (95%CI: 0.09; 0.13, I^2^ = 29.9%). Of the 293 cases, the cause of mortality was tumor progression in 83 cases, complications of the CT in 20, RT complications in 18, surgical complications in 15, hydrocephalus-related disorders in 10, infection in 7, and mortality in 59 cases had other underlying etiology. The cause of the mortality was not reported in 87 cases.

### 3.7. Treatment Strategies

**Figure 2:**
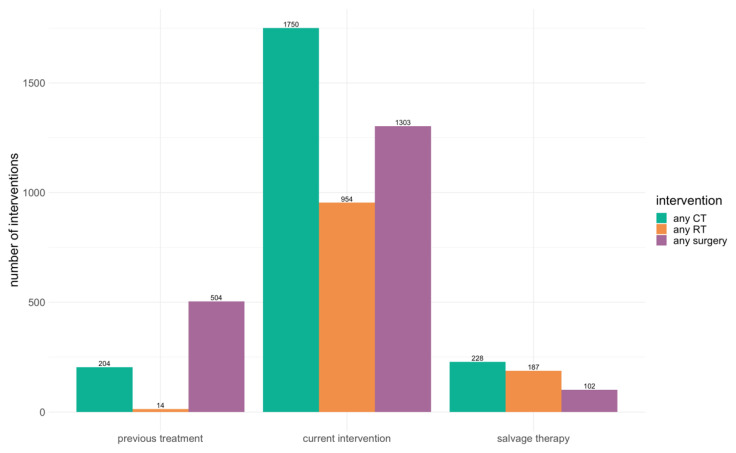
The treatment modalities used for OPG, according to the records of the patients.

Most of the articles reported the previous, current, and further salvage therapies. The details of the treatment strategies are presented in Figure 2 and Table 3.

### 3.8. Clinical Outcome

As visual acuity disruption is the most common presentation of the OPG, change in the vision of the patients was considered the clinical outcome of the treatment. The outcome of each treatment method is categorized as improved, not changed, and worsened. Due to the lack of sufficient data, improved and not changed visual outcomes were classified as one group. It is noteworthy that the outcomes of each treatment in one case were analyzed independently. For instance, if a patient received a CT in the first step, leading to exacerbation of the symptoms, and then subsequently underwent surgical intervention, leading to improvement of symptoms, the initial step was analyzed as the outcome of CT and the second step as the outcome of surgery. The overall pooled rate of the improved or not changed visual outcome is 0.75 (95%CI: 0.70–0.80, I^2^ = 53.4%, Q value = 121.68, *p* < 0.05). After the removal of outlier studies, due to the high between study heterogenicity, the rate of the outcome was 0.7793 (95%CI: 0.74; 0.81, I^2^ = 12.3%). The subgroup analysis also revealed no significant difference between the different treatment modalities (*p* = 0.92). Significance of the Egger test also was in favor of the publication bias and to compensate such issue, trim and fill method and Rucker limited meta-analysis were carried out which revealed 0.69 (95%CI: 0.62; 0.75, I^2^ = 58.7%, Q = 238.18, *p* = 0.0001) and 0.70 (95%CI: 0.61; 0.77, I^2^ = 53%, Q = 172.1, *p* = 0.0001) respectively. The details of the clinical outcome of each treatment strategy are described in Table 4.

**Figure 3:**
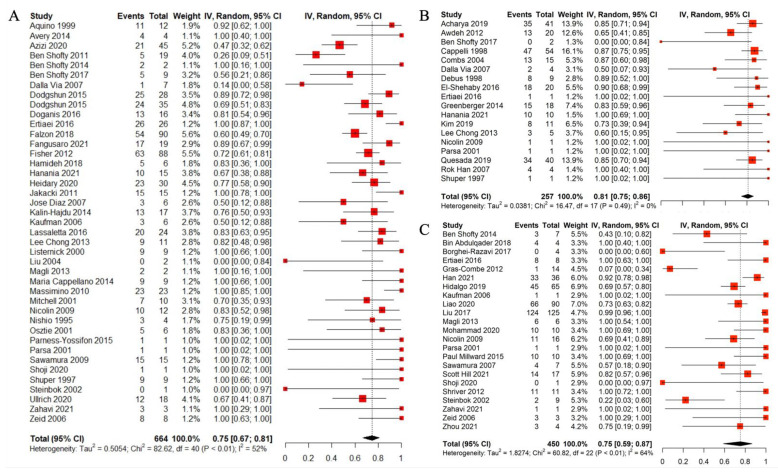
Forest plots for clinical outcome following each modality. (**A**) Chemotherapy, (**B**) radiotherapy, and (**C**) surgery [14,15,16,17,18,19,20,21,22,23,24,25,26,27,28,29,30,31,32,33,34,35,36,37,38,39,40,41,42,43,44,45,46,47,48,49,50,51,52,53,54,55,56,57,58,59,60,61,62,63,64,65,66,67,68,69,70,71,72,73,74,75,76,77,78,79,80,81,82,83,84,85,86,87,88,89,90,91,92,93,94,95,96,97,98,99,100,101,102,103,104,105,106,107,108,109,110,111,112,113,114,115,116,117,118].

The details of the clinical outcome following each modality are presented in the forest plots (Figure 3A–C show the outcome of CT, RT, and surgery, respectively).

### 3.9. Radiological Outcome

The classification and the analysis of the radiological outcome were performed the same as the clinical outcomes. After the treatment, the pooled rate of not changed or improved radiological outcome was 0.7167 (95%CI: 0.65; 0.77, I^2^ = 67.6%, Q = 274,82, *p*-value = 0.0001). Due to the I^2^ > 50%, removal of the 19 studies with outlier heterogenicity was done and the pooled estimate point was 0.7193 (95%CI: 0.67; 0.75, I^2^ = 21.8%, Q = 89.57, *p*-value = 0.057). The details of the radiological outcome of each treatment strategy are presented in Table 5.

**Figure 4:**
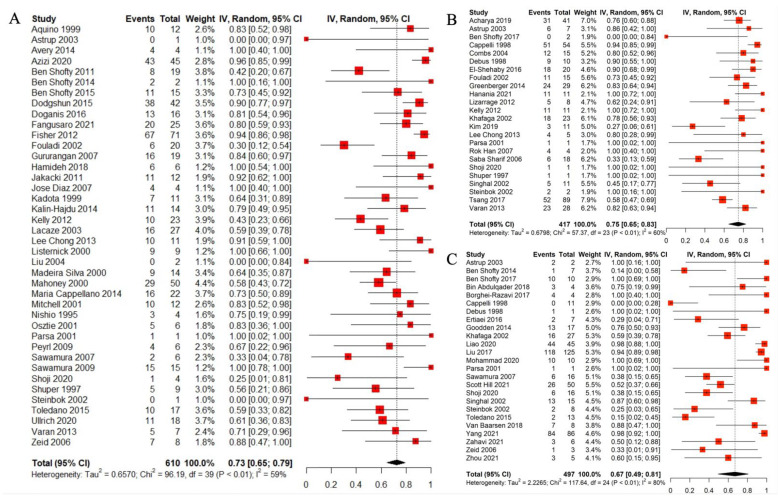
Forest plot for radiological outcome following each modality. (**A**) Chemotherapy, (**B**) radiotherapy, and (**C**) surgery [14,15,16,17,18,19,20,21,22,23,24,25,26,27,28,29,30,31,32,33,34,35,36,37,38,39,40,41,42,43,44,45,46,47,48,49,50,51,52,53,54,55,56,57,58,59,60,61,62,63,64,65,66,67,68,69,70,71,72,73,74,75,76,77,78,79,80,81,82,83,84,85,86,87,88,89,90,91,92,93,94,95,96,97,98,99,100,101,102,103,104,105,106,107,108,109,110,111,112,113,114,115,116,117,118].

The details of the radiological outcome following each modality are presented in the forest plots (Figure 4A–C show the outcome of CT, RT, and surgery, respectively).

## 4. Discussion

Although the OPGs are the most prevalent intrinsic optic nerve tumors, representing approximately 3–5% of all pediatric CNS malignancies, the overall outcome, either visually or radiologically, in each therapeutic modality is not investigated so far. These tumors are more common in children with NF-1 and arise more frequently during the first decade of life [2]. In this regard, our review demonstrated that almost half of the patients are known cases of NF-1, and the mean age of included patients was 8.3 years. Despite the fact that OPGs are mostly low-grade tumors, their behavior can be aggressive, and therapeutic approaches might face obstacles. In accordance with previously mentioned data, two-thirds of patients were classified as G1. Based on available data in the literature, out of 3360 cases of OPG, 348 deaths were recorded in which cause of mortality in 225 cases was declared. Tumor progression was the leading cause of death in these patients followed by procedure complications. A notable unfavorable predictive factor for mortality in OPG patients is believed to be the age of less than one year [102,121]. There are controversies regarding the role of NF-1 as a mortality predictive factor since in some studies patients with NF-1 had a higher progression-free survival (PFS) rate [82] while in others there was no statistically significant difference between patients with and without NF-1, respectively [122]. Considering a trend toward a better prognosis in NF-1 patients during the initial years of follow-up, this disparity fully vanished with time with the comparable overall survival (OS) rate between the two groups at the 15-year follow-up. The majority of NF-1 patient deaths occurred after tumor development and during the 10-year follow-up, demonstrating that these children would survive longer than the non-NF-1 patients. Cases of NF-1 usually died of an OPG rather than any other neurocutaneous syndromic problems, and the therapies they received, especially RT, seemed to have little impact on the long-term course of their condition [77,123]. A third prognostic factor is thought to be the existence of a diencephalic syndrome at the time of diagnosis, particularly once it is connected to leptomeningeal spread [122,124]. Based on available data, there were records of 165 cases of diencephalic syndrome in this study. Intracranial hypertension in patients with an OPG has been the subject of a few studies, but none of them looked at this symptomatology as a potential predictor of patient outcomes [1,125]. In terms of therapeutic approaches for these patients, observation, surgery, CT, and RT are all options for OPG management. The importance of each modality, along with a comparison to other modalities, are reviewed in this article. Though observation is a substantial option, there is a universal agreement that, in a considerable proportion of patients, OPG will pose a hazard to vision and, sometimes, become life-threatening secondary to the widely unpredictable natural history of these tumors [126]. It is important to carefully weigh treatment adverse effects against potential benefits, tumor growth prevention, and loss of critical capabilities, including vision [127]. OPG may have serious impacts on a child, but long-term survival is the issue that has the highest priority; thus, an evaluation of the long-term therapy’s side effects should be done before selecting a therapeutic modality.

### 4.1. Observation

A minority of youngsters with OPG do not need active intervention, according to certain retrospective evaluations [74,128]. As spontaneous regression was concerned in some other conditions [129], low-grade gliomas have been known to spontaneously regress as well [39,130]. Some investigators have questioned the justification for active care of OPG with surgery, CT, and/or RT in light of these data [127]. The question will probably go unanswered because no prospective research has compared observation with active intervention. There is currently agreement that individuals who show signs of neurological or visual impairment should be treated. However, children with NF-1 need to be given extra consideration since their OPG is reported to be more lethargic secondary to their primary disease [131]. On the other hand, many experts concur that the wait and watch strategy has a limited function in newborns and young children who do not have NF-1, especially when there is evidence of spread or a link with diencephalic syndrome [122,132]. It appears that intervention, particularly CT, has altered the natural history of this condition when compared to the historical series of patients with the diencephalic syndrome [2].

### 4.2. Radiotherapy

RT has been the cornerstone of the OPGs’ care for many years [133]. The objective of RT is to stop tumor development or regrowth, which has a risk of neurological impairment and vision loss. According to the method of analysis described before, the favorable radiological and visual outcome was achieved in 75% (95%CI 65–83) and 81% (95%CI 75–86) of patients who underwent different methods of RT, respectively. The lack of definitive follow-up prohibited us to perform a survival analysis on these patients. 5-year OS rates with RT in low-grade gliomas ranged from 79 to 96 percent, and 5-year PFS ranged from 48 to 100 percent, according to different studies [134,135]. With regard to the event-free survival rates reported in surgical and CT series, these findings show quite the same efficacy in RT as well. The 5-year OS rate and PFS rate in a group of patients who underwent surgical resection were 84.1 percent and 70.6 percent, respectively [59], whereas the OS rate in OPG patients who received CT was 95 percent [97]. However, since the demographic groupings are so dissimilar, comparisons between RT, surgery, and CT series are compromised. Young age and NF-1 are now understood to be the most significant predictors of OPG behavior, and OPGs are believed to be more aggressive in younger children [82,132]. Due to these unignorable disparities across the surgical, CT, and RT groups, the importance of such comparisons is therefore constrained. Studies have shown that postoperative RT focused on the chiasm is significantly more successful than surgery alone, leading to a lower percentage of treatment failure [136,137]. For these deeply located lesions, traditional RT is administered using parallel opposed fields, and the radiation volumes are typically large. This results in significant late side effects, including endocrinopathy, vasculopathy, as well as strokes, and neurocognitive disorders, especially in younger children [67].

The tissue that is intended to be destroyed and the adjacent normal tissue that is intended to be preserved in the treatment of benign lesions, including low-grade gliomas, are both of similar radiobiological type, meaning they are both late-responding tissues [26]. Therefore, it would be reasonable to assume that a fractionated course would not benefit from a single dosage in any way. To put it another way, a change in the fractionation pattern will not favorably cause more damage to the benign lesion than to the adjacent normal tissues [138]. Theoretically, stereotactic or conventional fractionated radiation would not be preferable to single-dose stereotactic radiosurgery. Radiation volume reduction, along with limiting the amount of normal brain tissue exposed to radiation while maintaining tumor control, was achieved by the introduction of 3D radiation therapy planning and delivery [134,139]. For OPG, the planned tumor volume typically extends 0.5 cm beyond the clinical target volume (CTV), and the agreed CTV typically extends 1.0 cm beyond the gross tumor volume. It is obvious that for these deeply positioned tumors, contemporary RT such as 3D conformal RT, intensity-modulated RT, stereotactic RT, and proton therapy are the preferred options. It is doubtful that current contemporary RT, especially proton therapy, can completely reduce the danger of vasculopathy, given the near closeness to the Willis circle [140]. Vasculopathy seems to be the most common side effect of RT in people with OPG [141,142]. Young age, a dosage of more than 50 Gy to the circle of Willis, NF-1, and previous surgery were additional known risk factors for the development of moyamoya as one of the most common vasculopathy in these patients [135]. Secondary tumor development is the other late unfavorable outcome following radiation. Following CNS radiation, the formation of secondary tumors is known to be risky [143]. A lower risk of secondary cancers following protons than following intensity-modulated RT has been shown by dosimetric comparison and biological modeling of probable radiation-induced toxicities [2]. However, the usefulness of such projections is constrained by ongoing advancements in radiation technology. According to the available data in this systematic review, one of the most prevalent complications of OPG, endocrinopathies were reported in 378 cases. Endocrinopathies, which are thought to be caused by pituitary gland damage, might develop from the tumor or its surgical treatment and cannot be primarily linked to radiation in OPG patients [108]. A variety of complicating circumstances, such as underlying NF-1, young age at diagnosis, large tumors, hormone impairment, or pre-existing hypothalamic damage, might make it difficult to assess radiation-associated neurocognitive impairments in patients with OPG. Only a small number of studies have used baseline testing and repeated post-radiation cognitive tests to comprehensively evaluate cognitive impairments. According to research by Merchant et al., most patients’ cognitive abnormalities are mild and predictable, and early age is linked to an increased likelihood of impairments [139]. Protons appear to offer long-term therapeutic advantages for children with OPGs as compared to photons, according to variations in radiation dose distributions that are shown by modeling changes in cognitive function [144].

### 4.3. Chemotherapy

The quest for alternative active therapy methods for OPG patients expanded as knowledge about the long-term problems linked to the use of radiation for treatment accumulated in the field of pediatric cancer. Patients who had failed RT were part of the earliest CT experiences. Rosenstock et al. reported the effective treatment of single-agent vincristine in a child with recurrent OPG following RT in the pre-computed tomography period [145]. A number of small-scale institutional investigations supported the ability of CT to slow the growth and even cause the shrinking of the low-grade gliomas [146,147]. The effectiveness of various drugs and combinations was proven by larger joint investigations [32,124]. The long-term effect of CT in patients with OPG, however, is yet unknown due to the distinctive design of CT investigations. Particularly, the effectiveness of CT in preventing vision deterioration has never been fully shown. The loss in visual acuity may be stopped, and eyesight may be stabilized by systemic administration of CT. According to the Fisher et al.’s study, almost one-third of children who received CT for NF-associated OPG had some improvement in their vision [32]. The main factor limiting the ultimate visual result, nevertheless, is pre-existing visual damage [31]. The thioguanine, procarbazine, lomustine, and vincristine (TPCV) regimen and the combination of carboplatin and vincristine are two of the most often utilized CT regimens [148,149]. Nevertheless, because of the greater risk of secondary neoplasms it carries, particularly for NF-1 individuals who are susceptible to secondary malignancies, the TPCV regimen continues to be the second line of treatment. Although ototoxicity restricts their usage, cisplatin and etoposide have also shown remarkable outcomes [73,150]. Additionally, individuals with OPG who received weekly vinblastine treatment had a PFS rate of 53.2% over a 5-year period, and 20% had improved visual acuity [89]. The inclusion or exclusion of NF-1 patients, variation in patient age and tumor status (newly diagnosed or recurrent), in addition to versatility in tumor site and response to treatment’s definitions, and progression interpretation all restrict comparisons across various CT regimens. The majority of OPG patients have been evaluated in studies in which their main focus delineated therapeutic effects of CT in low-grade glioma, although in general, no particular studies of this subgroup have been done. According to our analysis which is based on the available data, a favorable radiological outcome was achieved in 72% (95%CI 64–78) of OPG patients who underwent CT, while a favorable visual outcome was attained in 75% (95%CI 67–81) patients. Overall, CT is effective at stopping the tumor’s progression and vision loss in OPGs. CT offers stable or even lifelong tumor control in a large percentage of patients. Though in the absence of additional tumor advancement, the full response to CT is unusual, and the majority of patients will have signs of severe persisting magnetic resonance imaging abnormalities years after therapy. CT has different effects on OPG symptoms. With most regimens, CT has been shown to be beneficial in combating the diencephalic syndrome linked to hypothalamic/chiasmatic gliomas [151]. The visual reaction has only been documented in a small number of research, despite the fact that it should constitute a significant outcome measure. In Moreno et al.’s systematic review, the majority of patients have not altered visual symptoms, only a small percentage of patients have visual improvement, and there is no apparent correlation between visual response and radiological response [152]. Furthermore, the reliable assessment of visual parameters (visual acuity and visual fields), particularly in young children, is constrained by a variety of technical challenges [2]. Several concerns should be addressed before selecting a CT regimen. The inconsistent response criteria prevent comparative evaluation between regimens in terms of effectiveness. Vincristine-carboplatin and the TPCV regimen were compared in just one randomized research [149]. The response rate was comparable for both combinations. The PFS rate for patients receiving TPCV at 5 years was greater, but not in a significant way. The short- and long-term toxicity of these regimens may have an impact on treatment options. The occurrence of carboplatin hypersensitivity responses, which can approach 40% in certain series, limits the adoption of this regimen despite the fact that carboplatin is normally well tolerated [149,153,154]. In patients who received cisplatin-containing regimens, hearing loss was observed in 28% of cases, which raises concerns about the toxicity of the drug for patients who has visual deficits [150]. In the case of a benign tumor with a high chance of survival, prolonged exposure to alkylating drugs such as procarbazine, temozolomide, cyclophosphamide, and platinum compounds is debatable. In addition, there is a considerable risk of leukemia linked with the frequent use of the etoposide [155]. Studies are still being conducted to verify the encouraging outcomes shown with vinblastine, vinorelbine, bevacizumab, or either of its single-use formulations or the combination with irinotecan [118,156,157,158]. The majority of OPG patients will require many treatments. In the past, RT was the go-to salvage procedure when the initial round of CT had failed. CT is increasingly employed as an additional or follow-up therapy option, especially for young patients. According to certain findings, it is possible to administer CT repeatedly, and doing so does not negatively impact OPG patients’ visual outcomes [159]. The application of modern therapeutic modalities such as laser interstitial thermal therapy (LITT) for various brain pathologies is expanding [160,161]. A very recent report demonstrated promising results of LITT for two pediatric NF-1- associated, chemo-resistant, G1 pilocytic astrocytomas [162]. In order to confirm the LITT as a standard treatment for OPG, further studies are needed.

### 4.4. Surgery

The use of surgery in the treatment of OPG is not universally agreed upon. In addition to CSF diversion for related hydrocephalus, the surgical options are varied and include biopsy, partial/ full resection, and radical resection [163]. The long-term morbidities in the young half of OPG patients should be considered when physicians decide to select any therapeutic modalities. These morbidities include vision impairment, endocrinopathy, or other treatment-related issues that could have long-term psychological effects [164]. According to this systematic review and meta-analysis, a favorable outcome in surgery was achieved in 67% (95%CI 49–81) and 75% (95%CI 59–87), based on radiological and visual data, respectively. Due to the diffuse infiltrating character of OPG and the close proximity and involvement of eloquent brain regions, full excision is seldom possible. Total visual loss, and a tumor that is restricted to the optic nerve are the two conditions that allow for complete excision. Irradiation was once thought to be the only effective form of therapy for some tumors, most notably chiasmatic gliomas, and surgery was restricted to a biopsy for tissue diagnosis [133]. If there is no indication of cerebrospinal fluid blockage or a visual loss, initial surveillance is frequently used as a first course of action due to the unpredictable nature and observed stability of a high percentage of OPGs [68]. Based on anatomical distribution in imaging, the Dodge classification separates OPG into three groups: D1: solely the optic nerves, D2: chiasm involvement, with or without optic nerve involvement, and D3: involvement of the hypothalamus and/or other adjacent structures [119]. According to our systematic review of available data, the majority of patients were classified as D3, followed by D1 and D2, respectively. To support standardized, surgical evaluation of hypothalamic chiasmatic tumors, a revised anatomy-based staging system based on current neuroimaging should be created [165]. This system should include criteria to estimate the risk of the intensity of bilateral visual loss, surgical resectability, tumoral vascularity, tumor size, genetic profile, and age at assessment. To anticipate visual threats, the modified Dodge plan categorization was created [166]. The knowledge that many patients with these gliomas present with exophytic extension has led several neurosurgical teams to re-evaluate the surgical approach to these tumors during the past few decades, especially with the use of contemporary imaging. Additionally, it has been challenging to prove that radical surgery is beneficial in treating these large chiasmatic gliomas, and aggressive surgeries run the danger of damaging the visual system, hypothalamus, and vascular systems, leading to hypothalamic syndrome [57]. OPG does not develop backward and extends into the chiasm when it first presents without chiasmal involvement. Then, to avoid progression to the chiasm, surgical excision of an OPG is not required, especially for the intracranial component [131]. Sometimes, particularly when hydrocephalus develops as a result of third ventricular compression, hypothalamic or chiasmal gliomas may need to undergo surgical debulking [131]. Our systematic review demonstrated that 468 cases had increased intracranial pressure, which was the second most prevalent complication in OPG patients following the visual acuity impairment. Either debulking or resective surgery should be necessary in cases when one side’s eyesight is deteriorating in order to preserve the other side’s vision, manage hydrocephalus, and postpone RT [57]. Some common neurological manifestations of OPG are headache, nystagmus, developmental delay, and seizure which were reported in 185, 176, 67, and 39 patients. Where continuing development poses a hazard or worsens neurological function, debulking of large tumors is most likely to be beneficial [57]. Our results showed that strabismus occurred in 116 cases, while proptosis was reported in 250 cases. In patients with OPG-associated NF-1, either cosmetic reasons or treating corneal exposure could explain the rationale for surgical decompression restricted to partial excision of the intraorbital optic nerve [131]. In Steinbok et al. study, there was no link between the size of resection and the time to tumor progression, and patients who received restricted resections experienced fewer complications, particularly with regard to hypothalamic dysfunction [84]. Since the primary function of surgery is to offer tissue diagnosis and, if necessary, decompress the visual apparatus and/or the ventricular system, it may be inferred that there is no advantage in trying a radical excision of these tumors. Accordingly, Sawamura et al. study on optic pathway/hypothalamic gliomas demonstrated that in comparison with biopsy, due to a higher rate of postoperative complications in surgical removal of these tumors, initial resection surgery has limited advantages in these kinds of patients [75]. The use of surgery in the treatment of these malignancies is still debatable overall. Surgery is widely accepted as the primary type of therapy for unilateral optic nerve lesions linked to severe proptosis and/or total unilateral visual loss [1,49]. The relative benefits and drawbacks of debulking surgery vs restricted biopsy should be considered in detail in multidisciplinary conferences for other tumor types as it is unlikely that a randomized study would ever address these issues. Only in the case of abnormal imaging may a biopsy be indicated [57], and it is often useless for typical OPG in children with NF-1 [131]. The existence of a mass lesion blocking the foramen of Monro, resulting in hydrocephalus, is one specific feature that has to be considered. The requirement for CSF diversion in this situation could be avoided by surgical debulking [57]. Considering surgery as a management option later on in the course of the disease is possible because OPGs frequently reoccur [57]. The danger and impact of salvage surgery, however, have never been thoroughly assessed, and the majority of papers on the surgical therapy of OPGs have combined upfront debulking and salvage treatments [75]. Prospective comparative clinical studies that directly examine the effects of surgery and how it is best administered are scarce, which may not come as a surprise given the inherent difficulties of surgical trials in a rare pediatric illness.

### 4.5. Limitations and Future Research

Our systematic review has some limitations. Small sample sizes in studies that compare different therapeutic modalities for OPG treatment, heterogeneity in multiple parameters including tumor locations, patients’ age distribution who received treatment, patients lost to follow-up in many studies, lack of high-power studies, and insufficient power to adequately prove the efficacy or safety profile of different therapeutic modalities are all possible limitations of this systematic review. Moreover, in order to reduce the versatility between studies, we have considered all different types of surgical procedures, distinct CT regimens, and separate ways to deliver irradiation in OPG patients as surgery, CT, and RT, respectively. This assumption was made since available data were neither representative of actual therapeutic modalities nor provided an opportunity to conduct sub-group analysis. Additionally, since children’s visual assessments are notoriously recalcitrant, it might be obscure the results of visual acuity in pediatrics.

Further randomized controlled studies with larger patient sample sizes and adequate follow-up along with a precise definition of treatment modality on any single patient are needed to further validate the efficacy of each type of management in OPG patients.

## 5. Conclusions

To the best of our knowledge, this systematic review and meta-analysis is the first report on both visual and radiological outcomes of different therapeutic modalities in OPG patients. Although there was no statistically significant difference between different therapeutic modalities, due to the higher rate of remission in these methods in comparison with observation, the application of these therapeutic interventions in OPG patients results in a better outcome. Selecting the most appropriate therapeutic approach for OPG patients must be on an individual basis along with taking into account the multiple parameters. These items include complications and benefits of considered therapeutic modality particularly in the patient’s age range as well as the clinical and radiological presentations of the tumor.

## Figures and Tables

**Table 1:** The reported symptoms and their frequency among the patients with OPG.

Symptoms	Number of Patients
Decreased visual acuity	1656
Raised ICP	485
Endocrine disorder	378
Proptosis	250
Optic nerve disorder	232
Visual field defect	206
Neurological disorder	200
Headache	185
Nystagmus	176
Diencephalic syndrome	165
Strabismus	116
Other	87
Developmental delay	67
Other ophthalmologic disorder	49
Seizure	39
Cranial nerve involvement	11

**Table 2:** The reported post-intervention complications and their frequency among the patients with OPG.

Complications	Number of Patients
Neurological disorder	57
Seizure	15
Endocrine disorder	134
Electrolyte disturbance	94
Toxicity	334
Infection	37
Vasculopathy/hemorrhagic phenomenon	70
Ophthalmologic disorder	264
Hydrocephalus	48
Secondary tumor formation	44
Other	37

**Table 3:** The details of the treatment modalities of the OPG, according to the records of the patients.

Treatments Performed before the Study Design	Treatment	Number of Interventions
	Surgery	339
	Chemotherapy	201
	Radiotherapy	13
	Surgery + chemotherapy	2
	Surgery + chemotherapy + radiotherapy	1
	No specific report	90
**Treatments reported as the outcome of the studies**	**Treatment**	**Number of interventions**
	Chemotherapy	1338
	Surgery	853
	Radiotherapy	604
	Conservation	563
	Surgery + Chemotherapy	214
	Surgery + Radiotherapy	152
	Radiotherapy + Chemotherapy	114
	Surgery + Chemotherapy + Radiotherapy	84
**Salvage therapies**	**Treatment**	**Number of interventions**
	Chemotherapy	217
	Radiotherapy	169
	Surgery	91
	Other	37
	Radiotherapy + Chemotherapy	11
	Surgery + Radiotherapy	1

**Table 4:** The results of the meta-analysis, performed on the visual outcomes of the OPG patients.

Outcome	Analysis	Estimate Point	95% Confidence Interval	Tests of Heterogenicity		Number of Studies-Cases	Egger’s Test, *p*-Value
				I^2^	Q Value		
					*p* Value		
**Chemotherapy Visual Outcome**	Pooled rate	0.7453	0.66; 0.80	51.6%	82.6	41–664	0.0042
					0.001		
	outliers removed	0.7647	0.70; 0.81	27.8%	51.2	38	
					0.059		
	Trim and fill method *	0.6596	0.55; 0.74	59.6%	128.8	53	
					0.0001		
	Rucker’s limit meta-analysis *	0.6337	0.50; 0.74	51.6%	82.62		
					0.0001		
**Radiotherapy Visual Outcome**	Pooled rate	0.8110	0.74; 0.86	0%	16	18–257	0.3532
					0.49		
**Surgical intervention Visual Outcome**	Pooled rate	0.7532	0.58; 0.86	63.8%	60	23–450	0.6281
					0.0001		
	outliers removed	0.7535	0.65; 0.82	28.5%	26	20	
					0.11		

* Adjusted for publication bias.

**Table 5:** The results of the meta-analysis, performed on the visual outcomes of the OPG patients.

Outcome	Analysis	Estimate Point	95% Confidence Interval	Tests of Heterogenicity		Number of Studies-Cases	Egger’s Test, *p*-Value
				I^2^	Q Value		
					*p* Value		
**Chemotherapy Radiological** **Outcome**	Pooled rate	0.7256	0.64; 0.79	59.5	96.19	40–610	0.0704
					0.0001		
	outliers removed	0.7238	0.65; 0.78	27.5%	46.93	35	
					0.069		
**Radiotherapy Radiological** **Outcome**	Pooled rate	0.7470	0.64; 0.82	59.9%	57.3	24–417	0.1076
					0.0001		
	outliers removed	0.7570	0.68; 0.81	31.3%	29.1	21	
					0.08		
**Surgical intervention Radiological** **Outcome**	Pooled rate	0.6704	0.49; 0.80	79.6%	117.6	25–497	0.8007
					0.0001		
	outliers removed	0.5927	0.45; 0.71	44.9%	34.4	20	
					0.01		

## Data Availability

The data presented in this study are available in this article.
